# Novel Myopia Genes and Pathways Identified From Syndromic Forms of Myopia

**DOI:** 10.1167/iovs.17-22173

**Published:** 2018-01

**Authors:** D. Ian Flitcroft, James Loughman, Christine F. Wildsoet, Cathy Williams, Jeremy A. Guggenheim, for the CREAM Consortium

**Affiliations:** 1 Children's University Hospital and University College Dublin, Dublin, Ireland; 2 College of Sciences and Health, Dublin Institute of Technology, Dublin, Ireland; 3 Center for Eye Disease and Development, School of Optometry, University of California-Berkeley, Berkeley, California, United States; 4 Bristol Eye Hospital and Bristol University, Bristol, United Kingdom; 5 School of Optometry & Vision Sciences, Cardiff University, Cardiff, United Kingdom

**Keywords:** myopia, genetics, syndromic myopia, hyperopia, ametropia

## Abstract

**Purpose:**

To test the hypothesis that genes known to cause clinical syndromes featuring myopia also harbor polymorphisms contributing to nonsyndromic refractive errors.

**Methods:**

Clinical phenotypes and syndromes that have refractive errors as a recognized feature were identified using the Online Mendelian Inheritance in Man (OMIM) database. One hundred fifty-four unique causative genes were identified, of which 119 were specifically linked with myopia and 114 represented syndromic myopia (i.e., myopia and at least one other clinical feature). Myopia was the only refractive error listed for 98 genes and hyperopia and the only refractive error noted for 28 genes, with the remaining 28 genes linked to phenotypes with multiple forms of refractive error. Pathway analysis was carried out to find biological processes overrepresented within these sets of genes. Genetic variants located within 50 kb of the 119 myopia-related genes were evaluated for involvement in refractive error by analysis of summary statistics from genome-wide association studies (GWAS) conducted by the CREAM Consortium and 23andMe, using both single-marker and gene-based tests.

**Results:**

Pathway analysis identified several biological processes already implicated in refractive error development through prior GWAS analyses and animal studies, including extracellular matrix remodeling, focal adhesion, and axon guidance, supporting the research hypothesis. Novel pathways also implicated in myopia development included mannosylation, glycosylation, lens development, gliogenesis, and Schwann cell differentiation. Hyperopia was found to be linked to a different pattern of biological processes, mostly related to organogenesis. Comparison with GWAS findings further confirmed that syndromic myopia genes were enriched for genetic variants that influence refractive errors in the general population. Gene-based analyses implicated 21 novel candidate myopia genes (*ADAMTS18*, *ADAMTS2*, *ADAMTSL4*, *AGK*, *ALDH18A1*, *ASXL1*, *COL4A1*, *COL9A2*, *ERBB3*, *FBN1*, *GJA1*, *GNPTG*, *IFIH1*, *KIF11*, *LTBP2*, *OCA2*, *POLR3B*, *POMT1*, *PTPN11*, *TFAP2A*, *ZNF469*).

**Conclusions:**

Common genetic variants within or nearby genes that cause syndromic myopia are enriched for variants that cause nonsyndromic, common myopia. Analysis of syndromic forms of refractive errors can provide new insights into the etiology of myopia and additional potential targets for therapeutic interventions.

There is growing recognition that myopia represents a significant public health issue.^1–3^ In relation to developing preventive public health strategies and potential treatments, this makes understanding the etiology of myopia all the more important. There has been a long-running debate about whether myopia is predominantly genetic or environmental.^4,5^ The rising prevalence of myopia over recent decades has led to recognition that environmental effects must play an important role. Yet there remains strong evidence that genetic factors also make a significant contribution. The first clear evidence for a genetic role in myopia came from twin studies, with identical (monozygotic) twins showing a higher similarity (concordance) in refractive error than nonidentical (dizygotic) twins.^6,7^ Such studies have estimated heritability for myopia to be as high as 91%. Observed associations between parent refraction and that of their children may also have a genetic contribution.^8–11^ Conventional genetic linkage analysis studies revealed a number of potential loci for myopia-related genes, but the causative genes have mostly proved elusive.^12–14^

More recently, genome-wide association studies (GWAS) have attempted to identify genes associated with the risk of developing myopia. Early GWAS studies that focused on a handful of plausible candidate genes reported association between common gene variants and myopia or high myopia, using sample sizes of a few hundred participants; however, the results showed limited replication in later studies.^14,15^ In contrast, a move to sample sizes in the thousands, coupled with a much more systematic examination of common genetic variants across the whole genome, has proved more successful, with more than 100 gene loci identified and excellent concordance between studies.^16–19^ However, these loci together explain only a small percentage of the observed variation in refractive error (e.g., 2.3% in a study of children^20^ and 10% in a sample of adults^17^). The difference between the heritability estimates from twin and GWAS studies has been described as the “missing heritability” or “heritability gap” in myopia.^2,21^ This difference indicates either that twin studies have greatly overestimated the genetic contribution to refractive error, or a large number of refractive error–related genes have yet to be identified.

To date, one potential source of genetic variation in myopia has largely been overlooked, namely syndromic myopia, that is, myopia associated with at least one other medical condition. Many medical syndromes are genetic in origin, with clearly defined Mendelian inheritance patterns and positively identified genes. Some of the better-known forms of syndromic myopia are linked with genes that have a plausible role in myopia pathogenesis. For example Stickler's syndrome is most commonly associated with defects in the gene for collagen type II alpha-1 (*COL2A1*), which is expressed in the sclera, a structure that displays significant alterations in myopia.^22,23^ What has been lacking to date is a comprehensive analysis of the genetic basis of syndromic myopia and its potential broader relevance to refractive error development.

The primary aim of the present study was to examine the hypothesis that a proportion of human myopia results from polymorphisms in genes known to cause myopic syndromes. To achieve this aim, we compared the locations of identifiable syndromic myopia genes with data from two large GWAS studies that examined refractive errors and myopia (specifically, those carried out by CREAM^17^ and 23andMe^16^). Our second aim was to determine whether any specific biological processes that might be associated with the genetic control of refractive error could be identified from analyzing the functions of genes linked to syndromic myopia.

## Methods

### Identification of Genes Causing Syndromic Forms of Myopia

We identified clinical phenotypes that have refractive errors as a recognized feature using the Online Mendelian Inheritance in Man database (OMIM, Johns Hopkins University, Baltimore, MD, USA). OMIM is an expert-curated, comprehensive database of human genetic disorders and provides information about both the clinical features and specific causative genes where identified.

The entire OMIM database was downloaded in text format for off-line analysis following registration of this project with Johns Hopkins University (http://www.omim.org; in the public domain). Purpose-written software (predominantly written in AWK, a Unix scripting language) was used to process this text file to extract clinical syndromes that included any form of refractive error within the clinical features. The extraction of syndromes with refractive errors was performed on the basis of the phenotypic descriptors within OMIM. The latter are based on a clinical categorization from the wide variety of the cited papers for each condition, and therefore no consistent or specific dioptric thresholds could be derived. The list of terms used is shown in Table 1. The resulting set of phenotypic entries was then reviewed to ensure the reliability of the automated extraction process.

**Table 1:**
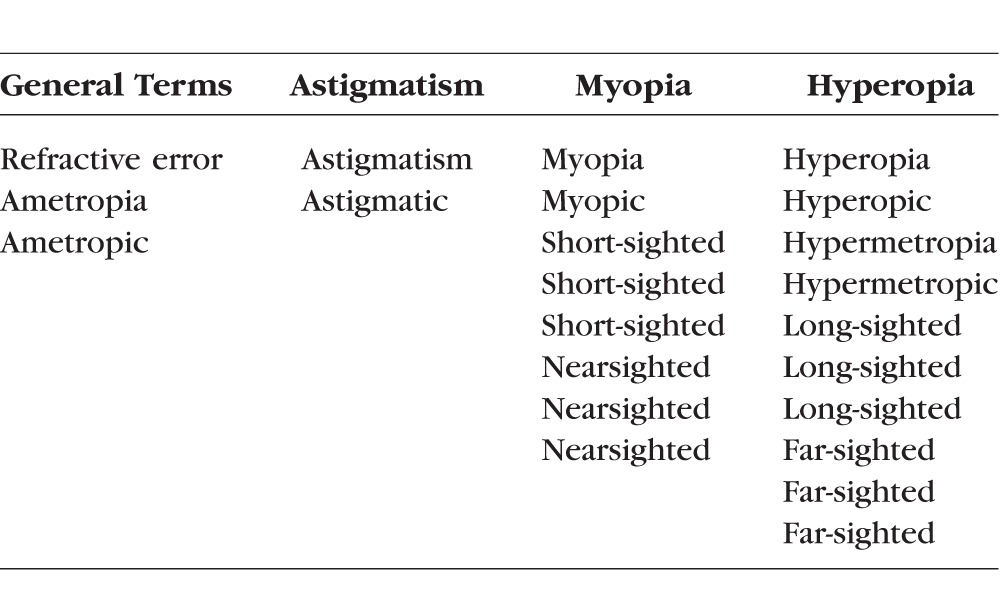
Search Terms Used to Detect Refractive Error Within OMIM Entries

Two additional OMIM database files (morbidmap and mim2gene.txt) were then used to determine if the phenotypes were associated with a specific gene and to extract the relevant NCBI Gene ID, Ensembl Gene ID, and the HGNC Approved Gene Symbol. The final dataset of OMIM-derived ametropia syndromes included OMIM entry number, clinical synopsis, type of refractive error, cytogenetic locus, Ensembl Gene ID, and HGNC (HUGO Gene Nomenclature Committee) nomenclature. All gene names referred to in this paper are the HGNC descriptors.

### Pathway Analysis: PANTHER, DAVID, REACTOME

Three different pathway analysis programs were used to search for pathways enriched for genes identified from the OMIM database. These were PANTHER (Protein Annotation Through Evolutionary Relationships, http://www.pantherdb.org/ [in the public domain], Version 11.0, released July 15, 2016),^24^ DAVID (Database for Annotation, Visualization and Integrated Discovery, Version 6.7, released January 27, 2010),^25^ and REACTOME Version 54 (release date September 30, 2015).^26^ The PANTHER Overrepresentation Test (release 20160715) was conducted using the GO Biological Process Ontology database released August 22, 2016, for human genes. Graphical representations of functional relationships were created using GOplot.^27^

### SNP-Based Comparison With CREAM GWAS Meta-Analysis for Refractive Error

We tested whether naturally occurring, common variants in the genes identified from the OMIM database were either more often or more strongly associated with refractive error than expected by chance. To do this, we took advantage of the meta-analysis of refractive error GWAS studies carried out by the CREAM Consortium.^17^ This CREAM GWAS meta-analysis included 37,382 individuals from 27 European studies and 8376 from 5 Asian studies. Our analysis was restricted to GWAS summary statistics for participants of European ancestry.

The CREAM study GWAS summary statistics provided a *P* value quantifying the association with refractive error for approximately 2.5 million autosomal genetic variants with a minor allele frequency (MAF) > 0.01 from phase 2 of the HapMap project.^28^ To identify single nucleotide polymorphisms (SNPs) in the vicinity of the genes identified by the OMIM search, the transcription start and stop sites of each of the 107 autosomal OMIM-derived myopia genes were retrieved and converted to genome build GRCh37 (hg19) coordinates. Twelve of the OMIM syndromic myopia genes were on chromosome X, which was not included in the CREAM GWAS meta-analysis; hence these X-linked genes could not be included in CREAM comparison analysis.

After updating the CREAM GWAS results to GRCh37 coordinates, CREAM SNPs positioned within ±50 kb of the 107 OMIM genes were selected, resulting in a list of 24,554 SNPs (note that the 50-kb flanking regions were included, since the majority of GWAS loci discovered to date are located in regulatory regions outside the coding region of genes^29^). Under the null hypothesis that SNPs in OMIM selected genes were neither more often nor more strongly associated with refractive error than chance level, 5% of the 24,554 SNPs (i.e., 1228) would be expected to attain a nominal level of statistical significance (*P* < 0.05). Similarly, under the null hypothesis, only a single SNP within the OMIM gene regions would be expected to reach an “experiment-wise” level of statistical significance of *P* = 4.4E-05 (i.e., 1/24,554).

### VEGAS: Gene-Based Comparison With CREAM GWAS Meta-Analysis for Refractive Error

We used VEGAS (Versatile Gene-Based Association Study)^30,31^ to carry out a gene-based analysis of whether naturally occurring, common variants in the genes identified from the OMIM database were either more often or more strongly associated with refractive error than expected by chance. The same set of 24,554 SNPs within ±50 kb of the 107 autosomal OMIM genes selected from the CREAM GWAS meta-analysis file, as described above, was submitted for analysis. VEGAS reported results for all 107 of these genes (*B3GLCT* and *P3H2* were listed in VEGAS as *B3GALTL* and *LEPREL1*, respectively).

### SNP-Based Comparison With 23andMe GWAS for Age of Onset of Myopia

A second, independent GWAS dataset was used to test whether naturally occurring, common variants in the genes identified from the OMIM database were either more often or more strongly associated with refractive error than expected by chance. The 23andMe company reported details of all 6141 genetic variants with *P* < 1E-04 from a GWAS using age of onset of myopia^16^ to assess approximately 7 million genetic variants (including variants on the X chromosome) in 45,000 participants of European ancestry. 23andMe imputed genotypes in their participants with the MaCH/minimac program, using the August 2010 release of the 1000 Genomes project as the reference panel. We downloaded the 04/08/2010 European reference panel data from the MaCH Web site (1000G.EUR.20100804.tgz), which listed a total of *N* = 11,914,767 variants. Of these 11.9 M variants, 87,598 were within ±50 kb of the 119 OMIM myopia genes. (Note that 23andMe used only ∼7.0 M for their GWAS, having excluded from the set of 11.9 M variants those with MAF < 0.005 and those that were poorly imputed; therefore our use of the full list of 87,598 variants is an overestimate of the true number of variants within the OMIM gene regions assessed by 23andMe and will be conservative). Under the null hypothesis of variants in OMIM syndromic myopia genes not being either more often or more strongly associated with age of onset of myopia than chance level, we would expect 0.01% of the 87,598 variants (i.e., 87,598/10^4^ = 9 variants) to attain *P* < 1.0E-04. Similarly, under the null hypothesis, only a single SNP within the OMIM gene regions would be expected to reach an experiment-wise level of statistical significance of *P* = 1.1E-05 (i.e., 1/87,598).

### Statistical Analysis

Pathway overrepresentation was tested using the methods implemented in PANTHER.^32^ A 1-sided binomial test was used to evaluate whether the number of genetic variants reaching a specified *P* value exceeded the number expected by chance, using the R statistics package (RStudio Team [2015], RStudio: Integrated Development for R, Boston, MA, USA).

## Results

A total of 219 clinical entities were identified within the OMIM database as conditions featuring ametropia, and in 167 cases at least one causative gene had been identified at the time of analysis. Of these 167 genetically defined, ametropia-featuring phenotypes, 162 were syndromic forms of refractive error, with at least one other phenotypic feature, and 5 were known Mendelian forms of high myopia. Myopia was the most commonly listed refractive error (*n* = 130), followed by hyperopia (*n* = 43) and astigmatism (*n* = 23). Myopia was the only type of refractive error described in 107 phenotypes. In 23 phenotypes, myopia was listed along with one or more other types of refractive error. Overall, 154 unique genes linked to various types of refractive error were identified within this group of conditions. The phenotypic subset associated with myopia (*n* = 130) was found to represent a set of 119 unique genes since 7 genes (*COL2A1*, *COL11A1*, *FBN1*, *LTBP2*, *POMT1*, *POMT2*, *POMGNT1*) had two or more associated phenotypes, leading to 11 duplicates. Hyperopia was associated with 42 unique syndromic genes, and astigmatism with 23 unique syndromic genes. Of the 119 myopia-associated genes, 107 were autosomal and 12 were on the X chromosome.

**Figure 1:**
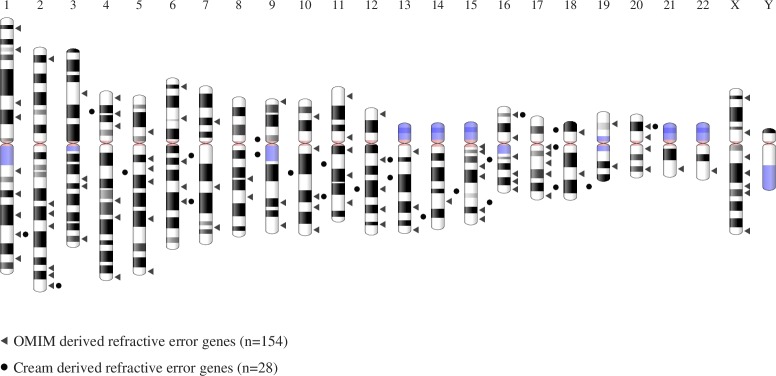
Chromosomal locations of all genes associated with syndromic ametropia in the OMIM database and the genes identified in the CREAM Consortium GWAS analysis.

A comparison of the chromosomal locations of the complete set of 154 refractive error–associated genes with the genes identified in the CREAM Consortium GWAS analysis^17^ is shown in Figure 1. The complete list of ametropia-associated genes identified within OMIM is given in Supplementary Table S1.

### Pathway Analysis

The biological processes associated with the 154 OMIM genes linked to refractive errors were examined using the PANTHER Overrepresentation Test, with Bonferroni correction for multiple tests. This approach estimates both the overrepresentation of a specific category of genes and the associated *P* value. For the complete group of ametropia genes, a wide range of biological processes was identified, as shown in Table 2. This table lists the identified biological process, the observed number of genes associated with that process, the expected number of such genes, the enrichment ratio, and the associated corrected *P* value. It includes all biological processes that were found to be statistically significant and where the level of gene overrepresentation was at least a factor of 10.

**Table 2:**
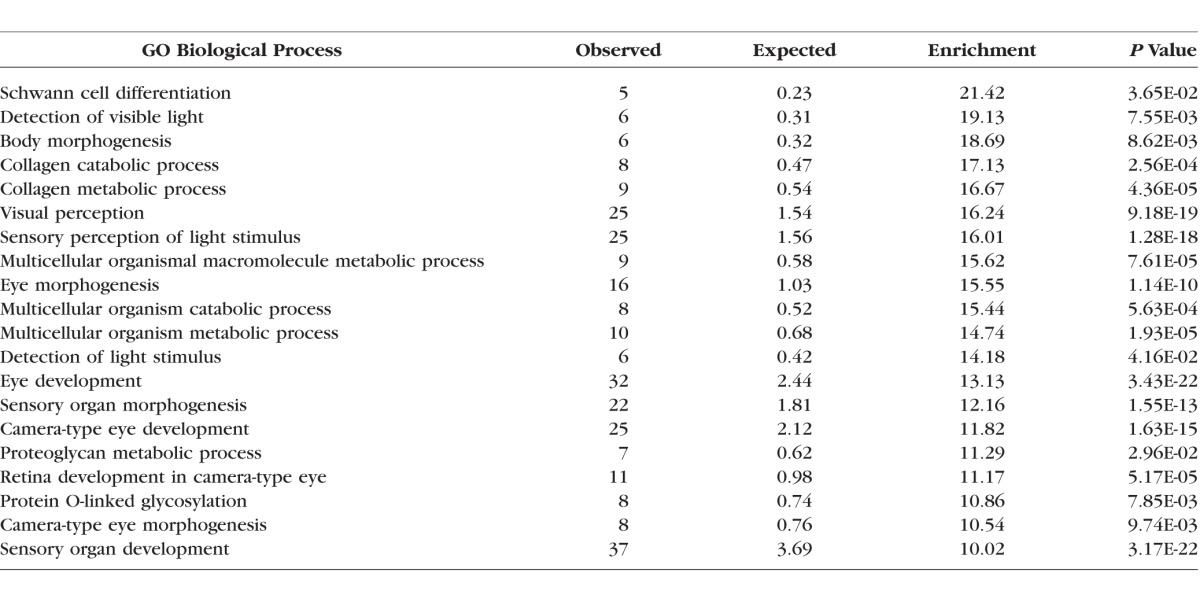
Biological Processes Overrepresented Among 154 OMIM Genes Known to Cause Syndromic Ametropia (PANTHER Analysis)

A similar analysis for genes specifically associated with myopia (rather than any type of ametropia) also revealed a wide range of biological processes (see Table 3). Several of these processes are highly plausible candidates for a role in the pathogenesis of myopia, such as collagen organization and metabolism, while others have not previously been associated with myopia development, such as protein mannosylation, glycosylation, and Schwann cell differentiation.

**Table 3:**
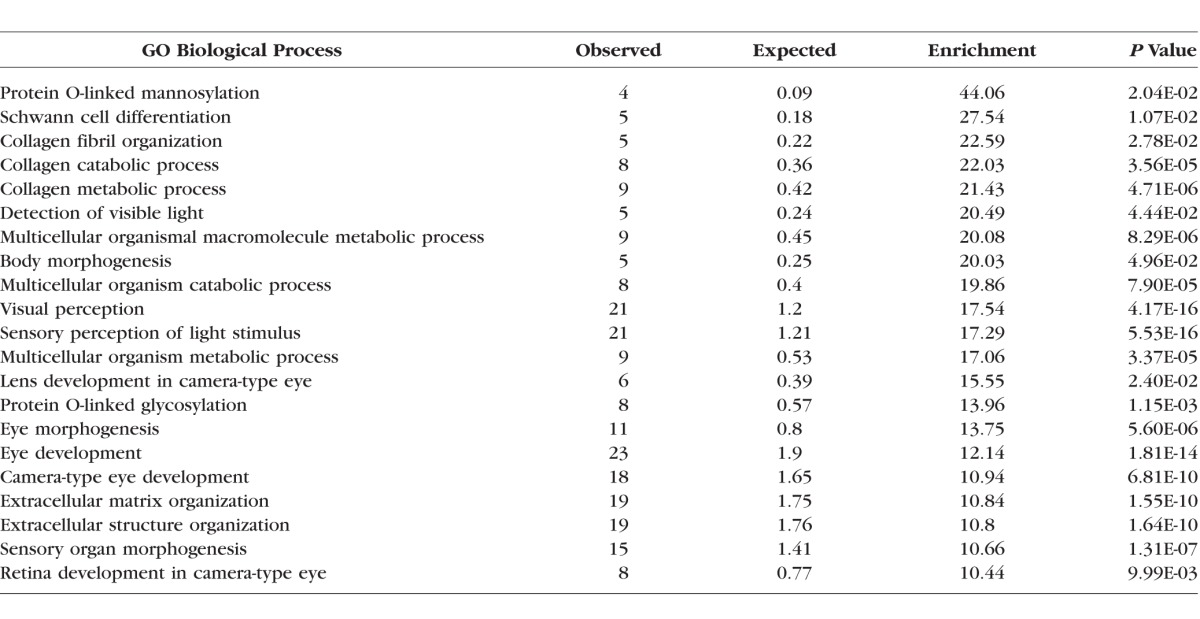
Biological Processes Overrepresented Among 119 OMIM Genes Known to Cause Syndromic Myopia (PANTHER Analysis)

**Figure 2:**
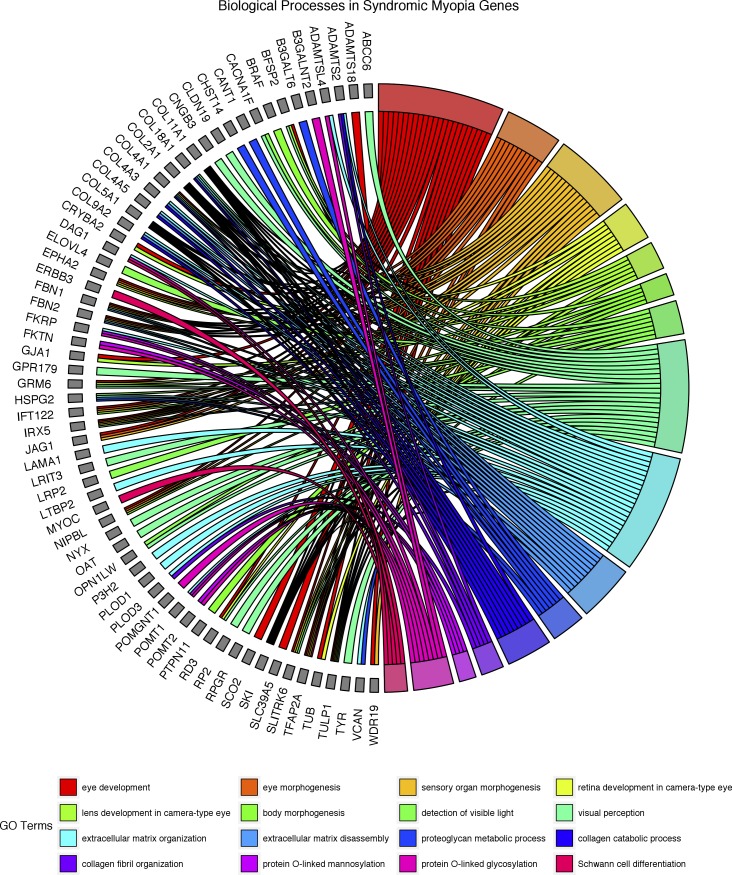
Diagram of overrepresented biological processes in syndromic myopia and the associated genes.

There is considerable overlap between the overrepresented biological processes found in myopia-related genes and the broader group of ametropia-related genes, as might be expected since myopic syndromes are the largest subgroup. Nevertheless, several processes either are unique to the myopic syndromes, or show greater overrepresentation when compared to the ametropic group. The four processes that are significantly overrepresented in myopia but not in the ametropia group are protein O-linked mannosylation (GO process: 0035269), collagen fibril organization (GO process: 0030199), lens development in camera-type eye (GO process: 0002088), and gliogenesis (GO process: 0042063). Thirteen processes are highly overrepresented (>10-fold) within the myopic syndromes to a greater degree than is observed within the ametropic syndromic genes: protein O-linked glycosylation (GO process: 0006493), Schwann cell differentiation (GO process: 0014037), collagen metabolism processes (GO processes: 0030574 and 0032963), visual processing (GO processes: 0009584, 0007601, 0050953), extracellular matrix organization and structure (GO processes: 0030198 and 0043062), and nonspecific metabolic processes (GO processes: 0044243, 0044259, 0044236). Figure 2 shows the individual myopia-related genes associated with these overrepresented biological processes and their links to those processes. As shown, several genes are linked to more than one biological process.

A different pattern of biological processes was identified in syndromes associated with hyperopia. There were just four processes showing a greater than 10-fold overrepresentation (*P* < 0.001 for each process) over that expected from an equally random sample of human genes: sensory organ morphogenesis, camera-type eye development, eye development, and sensory organ development. All four processes were also identified in myopic and ametropic syndromes, but with a lower level of overrepresentation. For syndromes that had astigmatism as a feature there were 23 unique genes, but no significant enrichment was found using the PANTHER analysis.

An analysis using DAVID identified enrichment of genes involved in extracellular matrix (ECM) receptor interaction (*P* = 5.4E-03), as well as those involving O-mannosyl glycan biosynthesis (*P* = 8.9E-03) and focal adhesion (*P* = 3.5E-02). Finally, pathway analysis using REACTOME identified enrichment for signaling by PDGF (platelet-derived growth factor; *P* = 6.3E-06), axon guidance (*P* = 2.8E-04), and integrin cell surface interactions (*P* = 2.1E-03).

### SNP-Based Comparison With CREAM GWAS Meta-Analysis for Refractive Error

**Figure 3:**
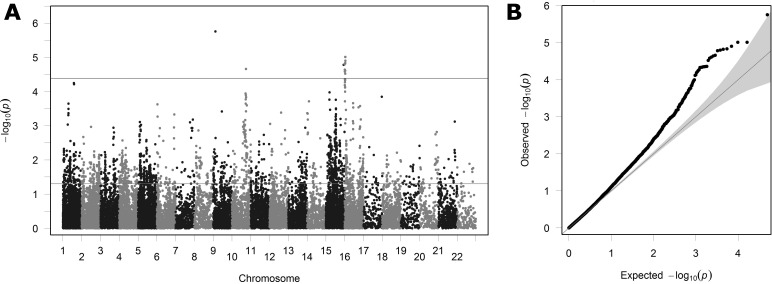
Manhattan plot (A) and QQ plot (B) showing the observed degree of association (y-axis; minus log_10_ of P value) in the CREAM Consortium GWAS for the 24,554 SNPs lying within 50 kb of the 107 autosomal OMIM derived myopia genes. The two horizontal lines in (A) indicate nominal (P = 0.05) and experiment-wise (P = 4.1E-05) level of statistical significance, respectively. The gray shaded region in (B) indicates the 95% confidence interval in which points would lie under the null hypothesis of no enrichment for SNPs associated with refractive error.

Figure 3 shows the level of association between genotype and refractive error observed in the GWAS meta-analysis of 37,382 European participants, carried out by the CREAM Consortium for the 24,554 SNPs lying within 50 kb of the 107 autosomal OMIM syndromic myopia genes. Of these 24,554 SNPs, 1717 exceeded the nominal level of statistical significance (*P* < 0.05), and 15 exceeded the experiment-wise significance threshold. These numbers are far higher than expected by chance (*P* < 2.2E-16 and *P* = 3.0E-13, respectively; binomial test), arguing strongly against the null hypothesis that the OMIM syndromic genes were not enriched for variants associated with refractive error. The QQ plot (Fig. 3B) also suggests that SNPs in the OMIM gene regions are consistently more strongly associated with refractive error than expected by chance (i.e., outside the 95% confidence intervals). A Manhattan plot (Fig. 3A) shows these strongly associated SNPs to be distributed across many genomic regions rather than all being clustered within one or two OMIM gene regions.

### Gene-Based Comparison With CREAM GWAS Meta-Analysis for Refractive Error

The above SNP-based comparison between genes identified by the OMIM search and the CREAM Consortium GWAS meta-analysis was carried out under the assumption that SNP associations were independent of each other. While this assumption is reasonable for distantly spaced SNPs, it will be violated for SNPs in the same region, since alleles are often inherited together on the same parental chromosome—a phenomenon known as linkage disequilibrium (LD). VEGAS analysis takes account of LD between SNPs in order to provide a gene-based assessment of the evidence for greater levels of association than expected by chance.

**Figure 4:**
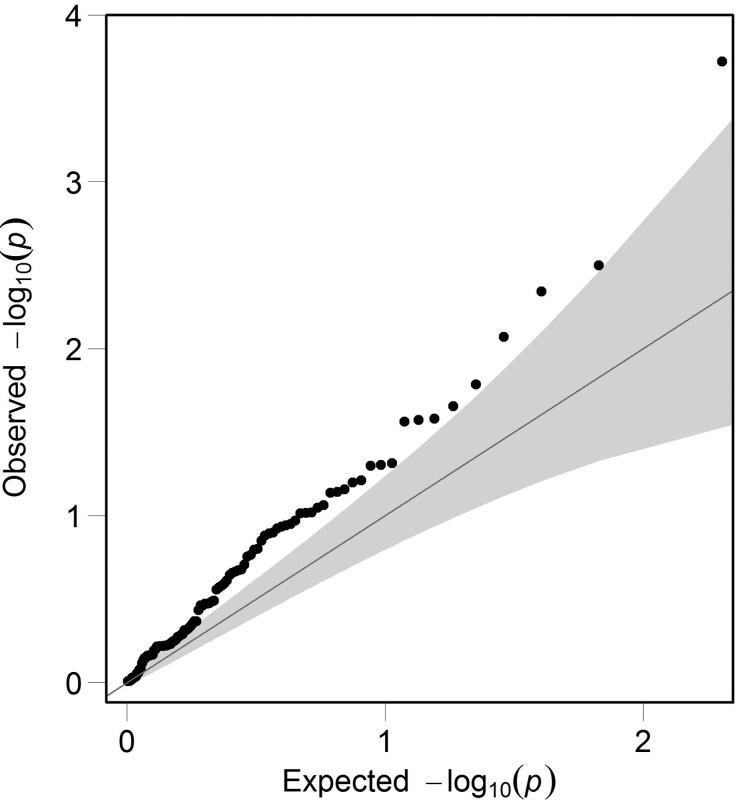
QQ plot for genes examined by VEGAS. The observed degree of association (y-axis; minus log_10_ of P value) in the VEGAS analysis for 107 autosomal OMIM derived myopia genes. The gray shaded region in the QQ plot indicates the 95% confidence interval in which points would lie under the null hypothesis of no enrichment within genes for SNPs associated with refractive error.

For the 24,554 SNPs examined above, VEGAS reported results for all 107 autosomal OMIM syndromic myopia genes. By chance, that is, under the null hypothesis that the OMIM genes were not enriched for SNPs associated with refractive error, approximately 5% of the 107 genes (i.e., 5 genes) would be expected to reach a nominal level of statistical significance. In contrast, VEGAS analysis identified 17 genes that also exceeded this threshold, but this was unlikely to have occurred by chance (*P* = 2.6E-05; binomial test). The OMIM-derived syndromic myopia genes that had greater than expected levels of enrichment for refractive error-associated SNPs in the VEGAS analysis are shown in Table 4. A QQ plot confirmed that the distribution of *P* values from the VEGAS analysis was enriched for strongly associated genes compared to that expected by chance (Fig. 4).

**Table 4:**
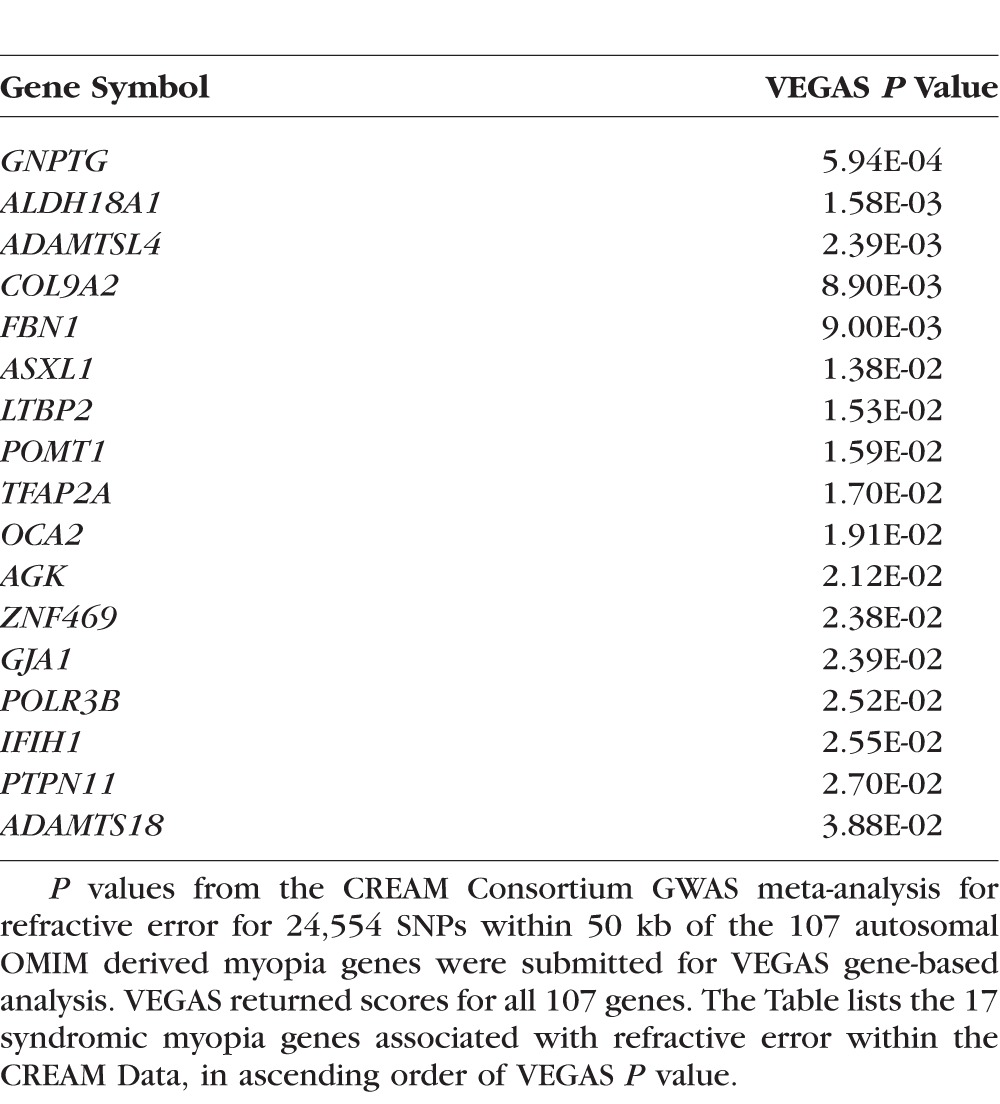
Results of the VEGAS Gene-Based Analysis

Kiefer et al.^16^ from 23andMe carried out a GWAS for age of onset of myopia, reporting 6141 genetic variants (SNPs and short insertions/deletions) that exceeded *P* < 1.0E-04 for association with myopia in their sample of 45,000 participants of European ancestry. However, we did not have information regarding which genetic variants were tested by Kiefer et al. and therefore we could not determine the precise number of variants within 50 kb of the 119 OMIM syndromic myopia genes. We estimated the maximum possible number of variants that could have been within 50 kb of the 119 OMIM syndromic myopia genes to be 87,598 (see Methods section for details). By chance, 0.01% of these 87,598 variants, that is, 9 variants, are expected to reach *P* < 1.0E-04. In fact, many more than this, 31 variants, exceeded this threshold (*P* < 4.3E-09; binomial test). These 31 variants were clustered near five of the OMIM syndromic myopia genes (Table 5). However, none of the 31 variants exceeded the experiment-wise threshold of *P* < 1.1E-05 for the 23andMe analysis. There was no overlap between the 5 OMIM-derived genes showing enrichment in the 23andMe GWAS for age at onset of myopia (Table 5) and the 17 genes showing enrichment in the VEGAS analysis of the CREAM GWAS for refractive error (Table 4).

**Table 5:**
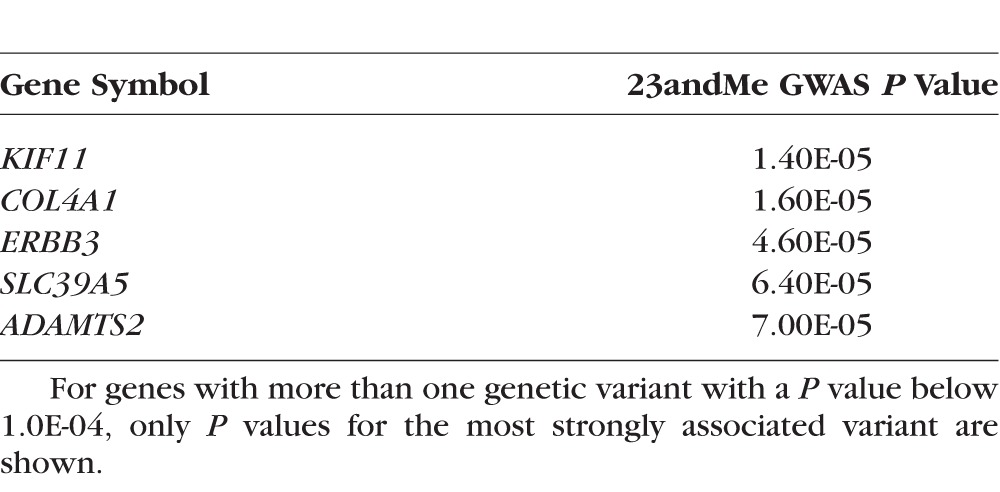
OMIM Syndromic Myopia Genes Showing Enrichment of Highly Associated Genetic Variants in the 23andMe GWAS for Age of Onset of Myopia

## Discussion

The genomic analysis of syndromic myopia described in this paper has identified a number of additional genes of potential relevance for myopia development in the general populations on which the CREAM and 23andMe studies were based. Of the 22 genes listed in Tables 4 and 5, only 1 (*SLC39A5*) is associated with one of the designated myopia (MYP) loci within OMIM, specifically the MYP24 locus. At the time of writing only four other MYP loci have had putative genes identified, these being MYP6 (gene: *SCO2*), MYP21 (gene: *ZNF644*), MYP22 (gene: *PRIMPOL*), and MYP23 (gene: *LRPAP1*). While none of these genes showed a significant association with refractive error in the VEGAS analysis of the CREAM GWAS dataset, *ZNF644* at the MYP21 locus is a member of the Krüppel C2H2-type zinc-finger protein family, and another member of this family, *ZNF469*, causes type 1 brittle cornea syndrome, which features blue sclera and myopia. Thus, the Krüppel C2H2-type zinc-finger protein family is of potential interest in terms of refractive error development. There is a notable lack of concordance in the VEGAS gene-based test results obtained using the CREAM GWAS summary statistics versus the 23andMe GWAS summary statistics. We speculate that this disparity is due to a combination of (1) the inherently low power of GWAS analyses at modest sample sizes to detect genetic association signals, and (2) the sparse genotype density of the 23andMe GWAS dataset, which provided very limited scope for VEGAS to harness multiple, independent signals from within the vicinity of individual genes. Had we been able to obtain access to the full set of GWAS summary statistics for the 23andMe study, as was the case for the CREAM study, we anticipate that the concordance of our VEGAS findings using the two datasets would have been higher (since there was high concordance for the 23andMe and CREAM GWAS analyses themselves: 16 of the 20 loci identified by 23andMe were confirmed by CREAM, and 14 of the 22 loci identified by CREAM were replicated by 23andMe^15^).

The clinical syndromes associated with the genes identified within this paper as having potential relevance for myopia are listed in Table 6. Mutations that cause rare disease syndromes tend to be rare themselves, because natural selection reduces the likelihood of their spread through the population. Such mutations usually affect the coding region of genes, resulting, for example, in errors of splicing or protein truncation,^33^ and therefore act deterministically to cause severe effects, irrespective of other genetic or environmental influences. By contrast, mutations/polymorphisms that lead to subtle phenotypic changes can become common in the population and most often affect noncoding, regulatory regions, resulting in variation in the expression level of a nearby gene.^34^ Nonetheless, the concept that polymorphisms in genes associated with severe clinical syndromes might contribute to common forms of myopia is supported by studies showing that genes causing syndromic high myopia can also cause high myopia as the sole clinical feature, for example, *NYX*^35^ and *GRM6*.^36^ That genes linked to severe phenotypes can be associated with common myopia has also been shown by the CREAM study. Table 7 lists the clinical syndromes from the OMIM database that are caused by genes for refractive error and myopia identified in the CREAM study. A total of 13 of the 28 genes identified by the CREAM and 23andMe studies are also known to cause defined, often severe, clinical syndromes affecting the eye and different organs. These data demonstrate that polymorphisms within the same gene can create both mild phenotypic variations in refractive error and severe clinical syndromes affecting the eye and other organs.

**Table 6:**
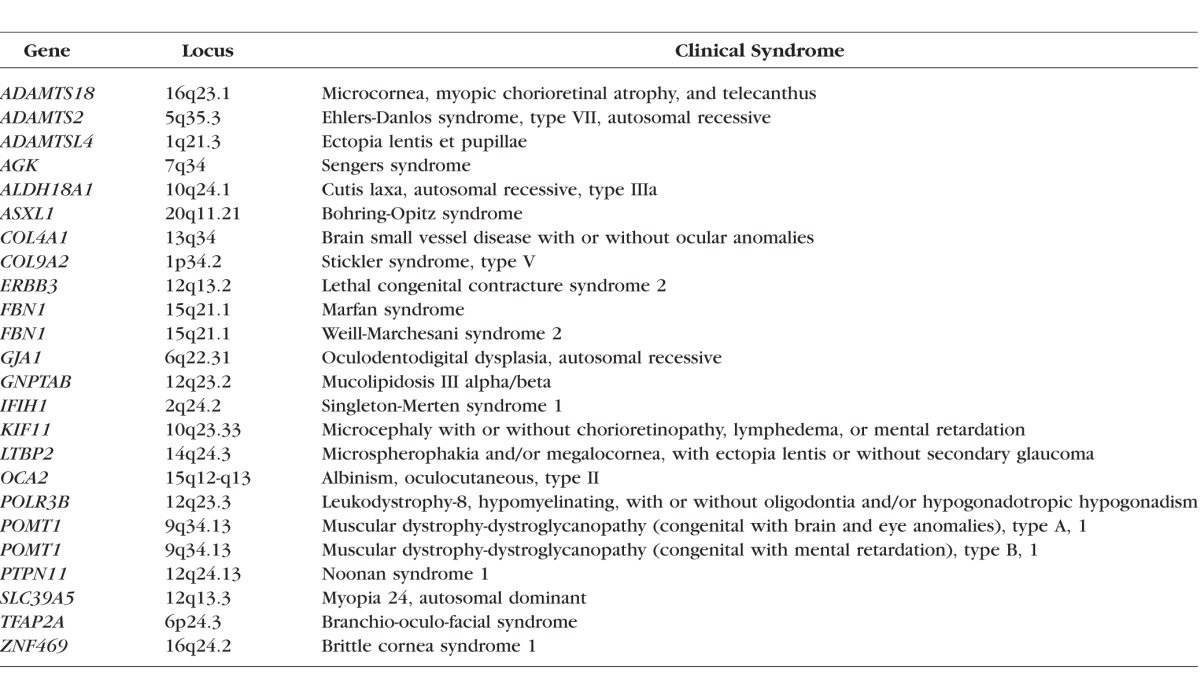
Syndromic Myopia Genes Linked to GWAS Analysis

**Table 7:**
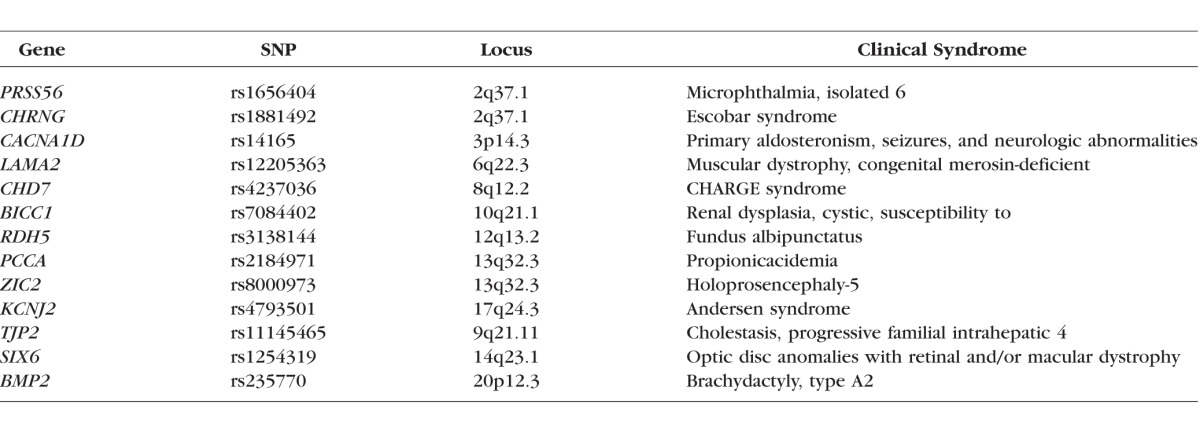
Clinical Syndromes Associated With Myopia Genes Identified by the CREAM Study

### Identification of Genetic Pathways Involved in the Etiology of Myopia

By their very nature, GWAS cannot determine whether a particular gene has a causal role in the condition being studied. Instead, when a genetic locus is identified by GWAS analysis, there may be one or more causative genes nearby, for example, within 100 kb. Often, the risk-determining polymorphism has a regulatory influence on the expression of one or more nearby genes.^34^ Nonetheless, for several of the genes identified by the CREAM Consortium and 23andMe, there is supporting evidence for a causal role in refractive error development; for example, *PRSS56* is associated with microphthalmia in humans and reduced eye size in knockout mice.^37,38^ *SIX6* is another gene involved in ocular morphogenesis.^39^ *RDH5* is involved in retinoic acid metabolism and all-trans retinoic acid has been identified as a potential intraocular signaling molecule in a mammalian model of myopia.^40^ *LAMA2* and *BMP2* are involved in ECM remodeling, and *BMP2* also appears to be an important intraocular signaling molecule for controlling eye growth.^41^ In contrast, all of the OMIM syndromic genes found to be statistically linked with myopia in the CREAM Consortium and 23andMe GWAS datasets (Tables 4, 5) have already been determined to have a direct role in producing myopia in humans.

The large number of genes identified from syndromic forms of myopia and other refractive errors also provided opportunity to study the biological mechanisms involved in refractive error by looking for patterns of overrepresentation of pathways within this set of genes. We analyzed GO terms overrepresented among OMIM syndromic ametropia and myopia genes (Tables 2, 3) to gain insight into underlying biological processes. GO terms are used to annotate sequences, genes, or gene products in biological databases, and represent a controlled vocabulary designed to facilitate the integration of information across species and biological disciplines. Although OMIM is not one of the databases directly contributing to the GO project, it is conceivable that researchers developing databases that do contribute to the GO project have utilized OMIM in their own work. This could potentially have led to some circularity in our pathway analysis, whereby one biological database (e.g., OMIM) is used to harness information from another database (e.g., the GO database). However, since OMIM is not a direct contributor to the GO project, we consider that any circularity would have little impact on the majority of our findings.

Many of the identified pathways (as shown in Fig. 2 and Tables 2, 3), such as connective tissue composition and ocular morphogenesis fit with prior hypotheses related to myopia development.^42,43^ Indeed, for the pathway analysis using REACTOME, all of the enriched pathways—namely, PDGF signaling, axon guidance, and integrin cell surface interactions—have already been implicated in myopia pathogenesis.^17,44–49^ Using other bioinformatics tools (PANTHER), several additional novel pathways were identified as being very overrepresented, including protein glycosylation, protein mannosylation, lens development, gliogenesis, and Schwann cell differentiation. This form of analysis also identified well-recognized pathways that may be involved in myopia development, such as those affecting collagen metabolism and other ECM components.

The largest OMIM analysis group related to genes linked to myopia (119 out of 154 ametropia genes). Thus the ametropia analysis is heavily weighted toward myopia, and there is considerable overlap in the biological processes that reached statistical significance when comparing myopia and all forms of refractive error. It is therefore doubtful that we can reliably distinguish pathways relating to ametropia per se from myopia. Nonetheless, in relation to whether the observed enrichment patterns in hyperopia and myopia make biological sense, the hyperopia-related genes showed a very different pattern of process enrichment from myopia-related genes, with emphasis on processes involved in early growth and organogenesis. This mirrors a fundamental difference between hyperopia and myopia in that hyperopia in adults usually represents a persistence of a refractive error that has existed since infancy, as compared to myopia, which, in the vast majority of cases, is acquired during childhood and early adult years. The smaller number of pathways found for hyperopia, as compared to myopia, is most likely to be the result of the smaller number of genes available for analysis. A sensitivity analysis of random subsamples of 46 out of the 119 myopes (to match the number of hyperopia-associated genes) showed a mean number of significant processes (*P* < 0.05 and >10× enrichment) reduced to 8.8 as compared to 22 with the full set of myopia genes (*n* = 119).

There are some identifiable, albeit speculative, links between these novel pathways and ocular development. Glycosylation and mannosylation are processes whereby macromolecules, such as proteins, are modified by the addition of simple sugars (glucose or mannose). In relation to glycosylation, high glucose levels in diabetes appear to promote myopia, perhaps by disrupting such pathways by nonenzymatic glycosylation.^50^ Primary glycosylation deficits (such as congenital disorder of glycosylation type Ia) cause significant brain dysfunction as well myopia, suggesting that a neuronal pathway may be involved.^51^ A similar association is seen with deficits in O-mannosylation, as demonstrated in Walker-Warburg syndrome (OMIM phenotype 253280). While the link with myopia remains unexplained, mannosylation is important for cell–matrix interactions and communication and therefore has a plausible role in the process by which neural elements within the eye influence the growth of its connective tissue components, including the sclera.^52^

There is experimental evidence that lens plays a role in eye growth. Experimental lensectomy in infant monkeys has been demonstrated to slow eye growth in monkeys less than 7.5 months of age, but not in older animals.^53^ It is therefore possible that genes influencing lens development may have an impact on early ocular growth and hence final refraction. Changes in lens thickness during later childhood have also been proposed to be an important factor in the development of myopia.^54^ Glial cells have received relatively little attention in relation to the development of refractive errors, but certainly merit more detailed attention. One of the CREAM-identified myopia risk genes (*PRSS56*) has recently been found to be expressed in retinal Müller cells and to have a role in refractive error development in mice (Paylakhi S, et al. *IOVS* 2017;58:ARVO E-Abstract 5635). Abnormal glial cell–derived myelination of retinal ganglion cells (i.e., myelinated retinal nerve fibers) is also associated with myopia development, though the mechanisms remain unexplained.^55^ Finally, within the eye Schwann cells are associated with the intrinsic neurons of the choroid,^56^ a structure implicated in ocular growth regulation.

Compared to studies of the genetic basis of myopia, hyperopia has received far less attention. A smaller number of syndromic hyperopia genes were identified in this analysis, but they were linked to different biological processes, principally involved in organogenesis. This fits in with the general clinical picture that most hyperopia is congenital and most myopia develops during postnatal growth.

### Role of X-Linked Genes

A significant number of genes associated with myopia were found on the X chromosome, which was not included in the CREAM and 23andMe analysis. Polymorphisms in the cone photopigment genes, which are located on the X chromosome, have been suggested as a cause for myopia.^57^ Might some GWAS studies have missed very important myopia-predisposing genes by excluding the X chromosome? The lack of any consistent differences in the prevalence in myopia between the sexes in adults makes this unlikely, as do the findings of a recently reported GWAS for myopia that did include the X chromosome.^19^ Nevertheless, the existence of X-linked forms of syndromic myopia does suggest that X-linked genes may have a role.

## Conclusions

In the present study, we screened polymorphisms located in and around genes known to cause rare genetic syndromes featuring myopia and found them to be overrepresented in GWAS studies of refractive error and myopia. This implies that while rare pathogenic mutations in these genes have profound, deterministic effects on the eye, more benign polymorphisms in and around these same genes may have subtle effects on ocular development and refractive error. Our identification of 21 novel genes (*ADAMTS18*, *ADAMTS2*, *ADAMTSL4*, *AGK*, *ALDH18A1*, *ASXL1*, *COL4A1*, *COL9A2*, *ERBB3*, *FBN1*, *GJA1*, *GNPTG*, *IFIH1*, *KIF11*, *LTBP2*, *OCA2*, *POLR3B*, *POMT1*, *PTPN11*, *TFAP2A*, *ZNF469*) and several novel pathways (mannosylation, glycosylation, lens development, gliogenesis, and Schwann cell differentiation) potentially involved in myopia is another small step toward explaining the missing heritability in refractive error.^2,21^ In contrast to myopia, hyperopia was found to be linked to a different pattern of biological processes, mostly related to organogenesis. Together with future efforts to increase the size and scale of GWAS projects, these discoveries will improve the ability to identify children most at risk of developing myopia for early treatment intervention.^58,59^

## Supplementary Material

Supplement 1
