# Supplementary material for: Novel Myopia Genes and Pathways Identified From Syndromic Forms of Myopia
Source: Invest Ophthalmol Vis Sci. 2018 Jan;59(1):338–48. doi: 10.1167/iovs.17-22173 (PMC5773233; doi:10.1167/iovs.17-22173)
Supplement: Supplement 1 [file iovs-58-14-16_s01.pdf]

Supplementary Table 1

Summary of all unique genes associated with refractive errors of all types (ametropia) that were identified from OMIM

Approved Gene ID	Alternative Gene Descriptors	Omim Gene Number	Ensembl ID	Linkage
ABCC6	ABCC6, ARA, ABC34, MLP1, PXE, GACI2	603234	ENSG00000091262	16p13.11
ADAMTS10	ADAMTS10, WMS1	608990	ENSG00000142303	19p13.2
ADAMTS17	ADAMTS17	607511	ENSG00000140470	15q26.3
ADAMTS18	ADAMTS18, MMCAT	607512	ENSG00000140873	16q23.1
ADAMTS2	ADAMTS2, NPI	604539	ENSG00000087116	5q35.3
ADAMTSL4	ADAMTSL4, TSRC1, ECTOL2	610113	ENSG00000143382	1q21.3
ADNP	ADNP, ADNP1, KIAA0784, HVDAS, MRD28	611386	ENSG00000101126	20q13.13
AGK	AGK, MULK, MTDPS10, CATC5, CTRCT38	610345	ENSG00000006530	7q34
ALDH18A1	ALDH18A1, PYCS, GSAS, ARCL3A	138250	ENSG00000059573	10q24.1
ASXL1	ASXL1, KIAA0978, BOPS, MDS	612990	ENSG00000171456	20q11.21
ATP6V0A2	ATP6V0A2, WSS, ARCL2A	611716	ENSG00000185344	12q24.31
B3GALNT2	B3GALNT2, MGC39558, MDDGA11	610194	ENSG00000162885	1q42.3
B3GALT6	B3GALT6, SEMDJL1, EDSP2	615291	ENSG00000176022	1p36.33
B3GAT3	B3GAT3, GLCATI	606374	ENSG00000149541	11q12.3
B3GLCT	B3GALTL, B3GTL, B3GLCT	610308	ENSG00000187676	13q12.3
BFSP2	BFSP2, CP49, CP47, CTRCT12	603212	ENSG00000170819	3q22.1
BRAF	BRAF, NS7	164757	ENSG00000157764	7q34
C12orf57	C12orf57, C10, TEMTYS	615140	ENSG00000111678	12p13.31
CACNA1F	CACNA1F, CSNB2, CORDX3, CSNB2A, AIED, OA2	300110	ENSG00000102001	Xp11.23
CANT1	CANT1, SCAN1, DBQD	613165	ENSG00000171302	17q25.3
CBS	CBS	613381	ENSG00000274276	21q22.3
CC2D2A	CC2D2A, KIAA1345, MKS6	612013	ENSG00000048342	4p15.32

CHMP1A	CHMP1A, PCOLN3, PRSM1, PCH8	164010	ENSG00000131165	16q24.3
CHRDL1	CHRDL1, VOPT, MGC1	300350	ENSG00000101938	Xq23
CHST14	CHST14, D4ST1, ATCS, EDSMC1	608429	ENSG00000169105	15q15.1
CLDN16	CLDN16, PCLN1, HOMG3	603959	ENSG00000113946	3q28
CLDN19	CLDN19, HOMG5	610036	ENSG00000164007	1p34.2
CNGB3	CNGB3, ACHM3, ACHM1	605080	ENSG00000170289	8q21.3
COL11A1	COL11A1, STL2	120280	ENSG00000060718	1p21.1
COL18A1	COL18A1, KNO1	120328	ENSG00000182871	21q22.3
COL2A1	COL2A1	120140	ENSG00000139219	12q13.11
COL4A1	COL4A1, POREN1, HANAC, ICH, BSVD, RATOR	120130	ENSG00000187498	13q34
COL4A3	COL4A3	120070	ENSG00000169031	2q36.3
COL4A5	COL4A5, ATS, ASLN	303630	ENSG00000188153	Xq22.3
COL5A1	COL5A1, EDSC	120215	ENSG00000130635	9q34.3
COL9A2	COL9A2, EDM2, STL5	120260	ENSG00000049089	1p34.2
COX7B	COX7B, LSDMCA2	300885	ENSG00000131174	Xq21.1
CRB1	CRB1, RP12, LCA8	604210	ENSG00000134376	1q31.3
CRYBA2	CRYBA2, CTRCT42	600836	ENSG00000163499	2q35
CTCF	CTCF, MRD21	604167	ENSG00000102974	16q22.1
DAG1	DAG1, DAG, MDDGC9, MDDGA9	128239	ENSG00000173402	3p21.31
ELOVL4	ELOVL4, ADMD, STGD2, STGD3, ISQMR, SCA34	605512	ENSG00000118402	6q14.1
EPHA2	EPHA2, ECK, ARCC2, CTPP1, CTPA, ARCC2, CTRCT6	176946	ENSG00000142627	1p36.13
ERBB3	ERBB3, LCCS2	190151	ENSG00000065361	12q13.2
ERCC4	ERCC4, XPF, FANCQ	133520	ENSG00000175595	16p13.12
ERCC6	ERCC6, CKN2, COFS1, CSB, ARMD5, UVSS1	609413	ENSG00000225830	10q11.23
ERCC8	ERCC8, CKN1, CSA, UVSS2	609412	ENSG00000049167	5q12.1
FAM111A	FAM111A, KIAA1895, KCS2, GCLEB	615292	ENSG00000166801	11q12.1
FBN1	FBN1, MFS1, WMS2, SSKS, GPHYSD2, ACMICD, ECTOL1	134797	ENSG00000166147	15q21.1

FBN2	FBN2, CCA, EOMD	612570	ENSG00000138829	5q23.3
FGD1	FGD1, FGDY, AAS, MRXS16	300546	ENSG00000102302	Xp11.22
FGFR3	FGFR3, ACH	134934	ENSG00000068078	4p16.3
FKBP14	FKBP14, EDSKMH	614505	ENSG00000106080	7p14.3
FKRP	FKRP, MDC1C, LGMD2I, MDDGA5, MDDGB5, MDDGC5	606596	ENSG00000181027	19q13.32
FKTN	FKTN, FCMD, CMD1X, LGMD2M, MDDGA4, MDDGB4, MDDGC4	607440	ENSG00000106692	9q31.2
FOXL2	FOXL2, BPES, BPES1, PFRK, POF3	605597	ENSG00000183770	3q22.3
GJA1	GJA1, CX43, ODDD, SDTY3, ODOD, HSS, AVSD3, HLHS1, CMDR, EKVP	121014	ENSG00000152661	6q22.31
GJC2	GJC2, GJA12, CX47, PMLDAR, HLD2, SPG44, LMPH1C	608803	ENSG00000198835	1q42.13
GNPTAB	GNPTAB, GNPTA	607840	ENSG00000111670	12q23.2
GNPTG	GNPTAG	607838	ENSG00000090581	16p13.3
GPR179	GPR179, GPR158L, GPR158L1, CSNB1E	614515	ENSG00000276469	17q12
GRM6	GRM6, MGLUR6, CSNB1B	604096	ENSG00000113262	5q35.3
HDAC8	HDAC8, WTS, MRXS6, CDLS5	300269	ENSG00000147099	Xq13.1
HSPG2	HSPG2, PLC, SJS, SJA, SJS1	142461	ENSG00000142798	1p36.12
IFIH1	IFIH1, MDA5, AGS7, SGMRT1	606951	ENSG00000115267	2q24.2
IFT122	IFT122, WDR10, CED1	606045	ENSG00000163913	3q21.3-q22.1
IRX5	IRX5, HMMS	606195	ENSG00000176842	16q12.2
JAG1	JAG1, AGS, AHD	601920	ENSG00000101384	20p12.2
KANSL1	KANSL1, KIAA1267, MSL1V1, KDVS	612452	ENSG00000275867	17q21.31
KCNH1	KCNH1, EAG, TMBTS, ZLS1	603305	ENSG00000143473	1q32.2
KCNV2	KCNV2, KV11.1, RCD3B	607604	ENSG00000168263	9p24.2
KDM5C	KDM5C, JARID1C, SMCX, DXS1272E, XE169, MRXSCJ	314690	ENSG00000126012	Xp11.22
KERA	KERA, CNA2	603288	ENSG00000139330	12q21.33
KIF11	KIF11, KNSL1, MCLMR	148760	ENSG00000138160	10q23.33

LAMA1	LAMA1, PTBHS	150320	ENSG00000101680	18p11.31-p11.23
LRIT3	LRIT3, FIGLER4, CSNB1F	615004	ENSG00000183423	4q25
LRP2	LRP2, DBS	600073	ENSG00000081479	2q31.1
LRPAP1	LRPAP1, A2MRAP, MYP23	104225	ENSG00000163956	4p16.3
LTBP2	LTBP2, LTBP3, GLC3D, MSPKA, WMS3	602091	ENSG00000119681	14q24.3
MAGEL2	MAGEL2, NDNL1, SHFYNG	605283	ENSG00000254585	15q11.2
MBD5	MBD5, KIAA1461, MRD1	611472	ENSG00000204406	2q23.1
MFRP	MFRP, MCOP5, NNO2	606227	ENSG00000259159	11q23.3
MIR184	MIR184, MIRN184, KTCNCT, EDICT	613146	ENSG00000207695	15q25.1
MYOC	MYOC, TIGR, GLC1A, JOAG, GPOA	601652	ENSG00000034971	1q24.3
NBAS	NBAS, NAG, SOPH, ILFS2	608025	ENSG00000151779	2p24.3
NDN	NDN	602117	ENSG00000182636	15q11.2
NDUFB11	NDUFB11, LSDMCA3	300403	ENSG00000147123	Xp11.23
NIPBL	NIPBL, CDLS1	608667	ENSG00000164190	5p13.2
NMNAT1	NMNAT1, NMNAT, PNAT1, LCA9	608700	ENSG00000173614	1p36.22
NOG	NOG, SYM1, SYNS1	602991	ENSG00000183691	17q22
NSD1	NSD1, ARA267, STO, SOTOS1	606681	ENSG00000165671	5q35.2-q35.3
NYX	NYX, CSNB1A, NBM1	300278	ENSG00000188937	Xp11.4
OAT	OAT, GACR	613349	ENSG00000065154	10q26.13
OCA2	OCA2, P, PED, D15S12, BOCA, EYCL3, HCL3, SHEP1	611409	ENSG00000277361	15q12-q13
OPN1LW	OPN1LW, RCP, CBP, CBBM	300822	ENSG00000102076	Xq28
OPTN	OPTN, GLC1E, FIP2, HYPL, NRP, ALS12	602432	ENSG00000123240	10p13
P3H2	P3H2, LEPREL1, MCVD	610341	ENSG00000090530	3q28
PCNT	PCNT, PCNT2, KEN, SCKL4, MOPD2	605925	ENSG00000160299	21q22.3
PIEZO2	PIEZO2, FAM38B, DA5, DA3, MWKS	613629	ENSG00000154864	18p11.22-p11.21

PIGT	PIGT, NDAP, PNH2, MCAHS3	610272	ENSG00000124155	20q13.12
PIK3R1	PIK3R1, GRB1, AGM7, SHORT, IMD36	171833	ENSG00000145675	5q13.1
PLOD1	PLOD1, LH1, LLH, EDS6	153454	ENSG00000083444	1p36.22
PLOD3	PLOD3, LH3	603066	ENSG00000106397	7q22.1
POLR1C	POLR1C, RPA39, RPA40, RPAC1, RPA5, TCS3, HLD11	610060	ENSG00000171453	6p21.1
POLR3B	POLR3B, RPC2, C128, HLD8	614366	ENSG00000013503	12q23.3
POMGNT1	POMGNT1, MEB, MDDGA3, MDDGB3, MDDGC3	606822	ENSG00000085998	1p34.1
POMT1	POMT1, MDDGA1, MDDGB1, MDDGC1	607423	ENSG00000130714	9q34.13
POMT2	POMT2, MDDGA2, MDDGB2, MDDGC2	607439	ENSG00000009830	14q24.3
PQBP1	PQBP1, NPW38, SHS, MRX55, MRXS3, RENS1, MRXS8	300463	ENSG00000102103	Xp11.23
PRDM5	PRDM5, BCS2	614161	ENSG00000138738	4q27
PRIMPOL	CCDC111, MYP22	615421	ENSG00000164306	4q35.1
PRSS56	PRSS56, MCOP6	613858	ENSG00000237412	2q37.1
PTEN	PTEN, MMAC1, GLM2, CWS1	601728	ENSG00000171862	10q23.31
PTPN11	PTPN11, PTP2C, SHP2, NS1, JMML, METCDS	176876	ENSG00000179295	12q24.13
RAB28	RAB28, CORD18	612994	ENSG00000157869	4p15.33
RD3	RD3, LCA12, C1orf36	180040	ENSG00000198570	1q32.3
RP2	RP2	300757	ENSG00000102218	Xp11.23
RPGR	RPGR, RP3, CRD, RP15, COD1, CORDX1	312610	ENSG00000156313	Xp11.4
RS1	RS1, XLRS1	300839	ENSG00000102104	Xp22.13
SCO2	SCO2, CEMCOX1, MYP6	604272	ENSG00000130489	22q13.33
SKI	SKI, SGS	164780	ENSG00000157933	1p36.33
SLC39A5	SLC39A5, MYP24	608730	ENSG00000139540	12q13.3
SLC6A8	SLC6A8, CRTR, CCDS1	300036	ENSG00000130821	Xq28
SLITRK6	SLITRK6, DFNMYP	609681	ENSG00000184564	13q31.1
SMAD4	MADH4, DPC4, SMAD4, JIP, MYHRS	600993	ENSG00000141646	18q21.2
SMARCAL1	SMARCAL1, HARP, SIOD	606622	ENSG00000138375	2q35

SMS	SMS, SRS, MRSR	300105	ENSG00000102172	Xp22.11
SOBP	SOBP, JXC1, MRAMS	613667	ENSG00000112320	6q21
SRCAP	SRCAP, SWR1, KIAA0309, FLHS	611421	ENSG00000080603	16p11.2
TBC1D24	TBC1D24, KIAA1171, FIME, EIEE16, DOORS, DFNB86, DFNA65	613577	ENSG00000162065	16p13.3
TBC1D7	TBC1D7, PIG51, TBC7, MGCPH	612655	ENSG00000145979	6p24.1
TCF4	TCF4, SEF2, ITF2, PTHS, FECD3	602272	ENSG00000196628	18q21.2
TEAD1	TEAD1, TCF13, REF1	189967	ENSG00000187079	11p15.3-p15.2
TFAP2A	TFAP2A, AP2TF, BOFS	107580	ENSG00000137203	6p24.3
THOC6	THOC6, FSAP35, BBIS	615403	ENSG00000131652	16p13.3
TIMM8A	TIMM8A, DFN1, DDP, MTS, DDP1	300356	ENSG00000126953	Xq22.1
TMEM98	TMEM98, NNO4	615949	ENSG00000006042	17q11.2
TRAPPC11	TRAPPC11, C4orf41, LGMD2S	614138	ENSG00000168538	4q35.1
TRIM37	TRIM37, MUL, KIAA0898	605073	ENSG00000108395	17q22
TRPV4	TRPV4, VROAC, HMSN2C, CMT2C, SPSMA, SSQTL1, SMAL, BCYM3	605427	ENSG00000111199	12q24.11
TUB	TUB, RDOB	601197	ENSG00000166402	11p15.4
TUBB3	TUBB3, TUBB4, CFEOM3A, CDCBM1	602661	ENSG00000258947	16q24.3
TULP1	TULP1, RP14, LCA15	602280	ENSG00000112041	6p21.31
TYR	TYR, SHEP3, CMM8, OCA1A, ATN	606933	ENSG00000077498	11q14.3
UBE3A	UBE3A, ANCR	601623	ENSG00000114062	15q11.2
UBE3B	UBE3B, BPIDS, KOS	608047	ENSG00000151148	12q24.11
VCAN	VCAN, CSPG2, WGN, WGN1, ERVR	118661	ENSG00000038427	5q14.2-q14.3
VPS13B	VPS13B, KIAA0532, COH1	607817	ENSG00000132549	8q22.2
VSX1	VSX1, RINX, PPCD1, PPD, KTCN1, CAASDS	605020	ENSG00000100987	20p11.21
WDR19	WDR19, SRTD5, ATD5, NPHP13, CED4	608151	ENSG00000157796	4p14

ZNF408	ZNF408, EVR6, RP72	616454	ENSG00000175213	11p11.2
ZNF469	ZNF469, KIAA1858, BCS1	612078	ENSG00000225614	16q24.2
ZNF644	ZNF644, MYP21	614159	ENSG00000122482	1p22.2
ZSWIM6	ZSWIM6, KIAA1577, AFND	615951	ENSG00000130449	5q12.1
